# Supplementary material for: N-acetyl-glucosamine primes Pseudomonas aeruginosa for virulence through a type IV pili/cAMP-mediated morphology transition
Source: Nat Commun. 2025 Oct 24;16:9405. doi: 10.1038/s41467-025-64071-0 (PMC12552656; doi:10.1038/s41467-025-64071-0)
Supplement: Supplementary file 1 — Supplementary Information [file 41467_2025_64071_MOESM1_ESM.pdf]

Supplementary figures to

N-acetyl-glucosamine primes *Pseudomonas aeruginosa* for virulence through a type IV pili/cAMP-mediated morphology transition

Jing Chen[1*], Guiying Lin[1, 2, 3], Kaiyu Ma[1], Yunxue Guo [4], Zi Li[1], Xiaoxue Wang[4, 5], Dominique Ferrandon[1,2,3*]

1 Sino-French Hoffmann Institute, School of Basic Medical Sciences, Guangzhou Medical University, Guangzhou, China.
2 Université de Strasbourg, Strasbourg, France.
3 Modèles Insectes de l'Immunité Innée, UPR 9022 du CNRS, Strasbourg, France.
4 South China Sea Institute of Oceanology, Chinese Academy of Sciences, Guangzhou, China.
5 Key Laboratory of Tropical Oceanography, South China Sea Institute of Oceanology, Chinese Academy of Sciences, Guangzhou, China

*Correspondence: chenjing_1127@qq.com; D.Ferrandon@unistra.fr

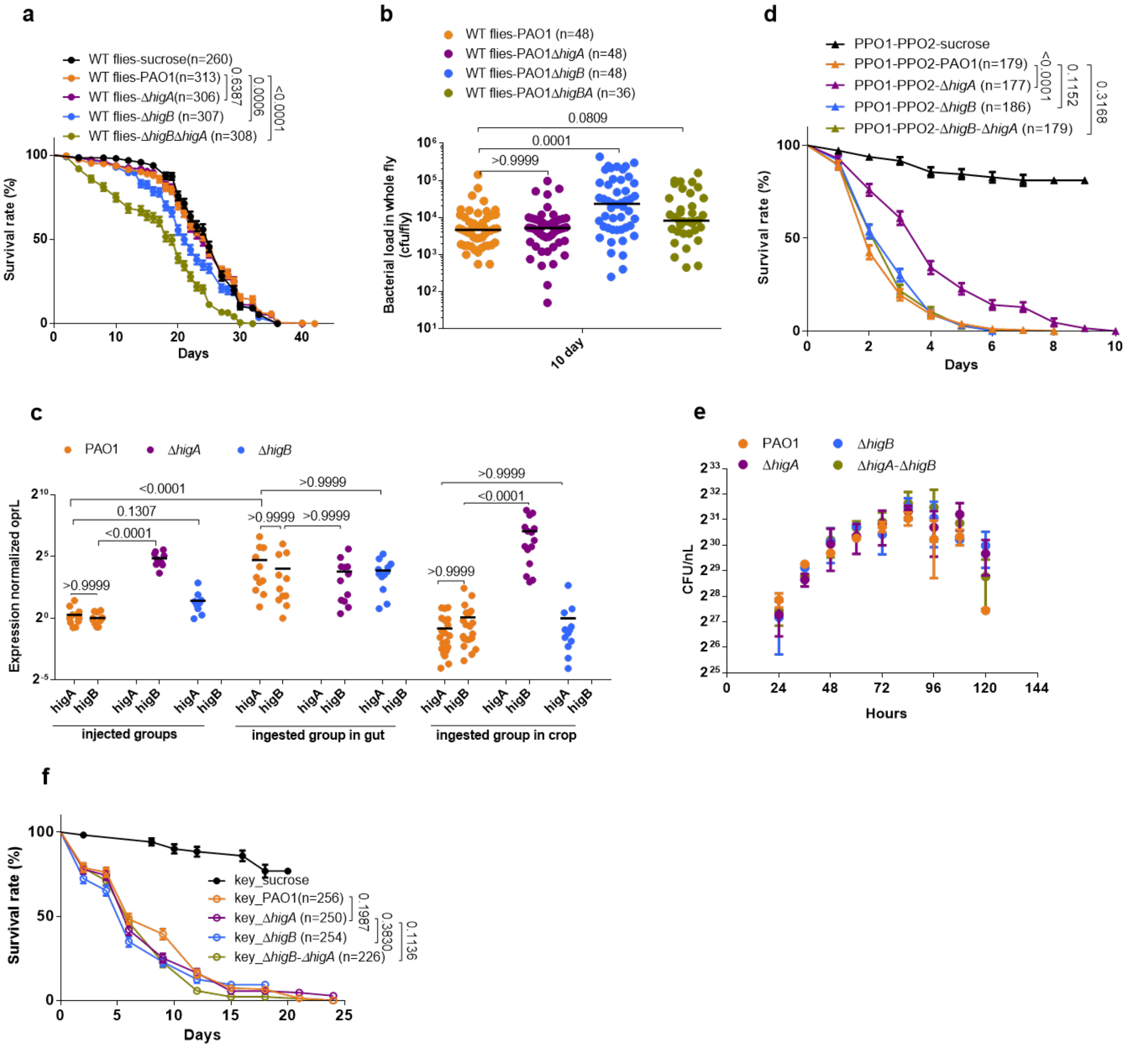

Supplementary Figure 1. Pathogenesis of a set of *higBA* mutants in *P. aeruginosa* latent infection

a Survival of flies challenged with a set of *higBA* mutants. **b** Bacterial load corresponding to (a). **c** Transcription level of *higBA* operon in different infection routes. **d** Survival of melanization-deficient flies challenged with a set of *higBA* mutants. **e** Growth characteristics of a set of *higBA* mutants *in vitro*. **f** Survival of *key* flies challenged with a set of *higBA* mutants. Bars represent standard error of the mean (a, d, f, e), median (b), mean (c). Experiments were repeated three times (a-d, f; pooled data; c: injected groups (n=8), ingested group in gut (n=12), ingested group in crop (n=22 for PAO1 and Δ*higA*, n=13 for Δ*higB*) and twice (e, representative data shown, n=2-6). Statistical analysis was done by Logrank (Mantel-Cox test) in (a, d, f), by Kruskal-Wallis with Dunn's post-hoc test in (b-c).

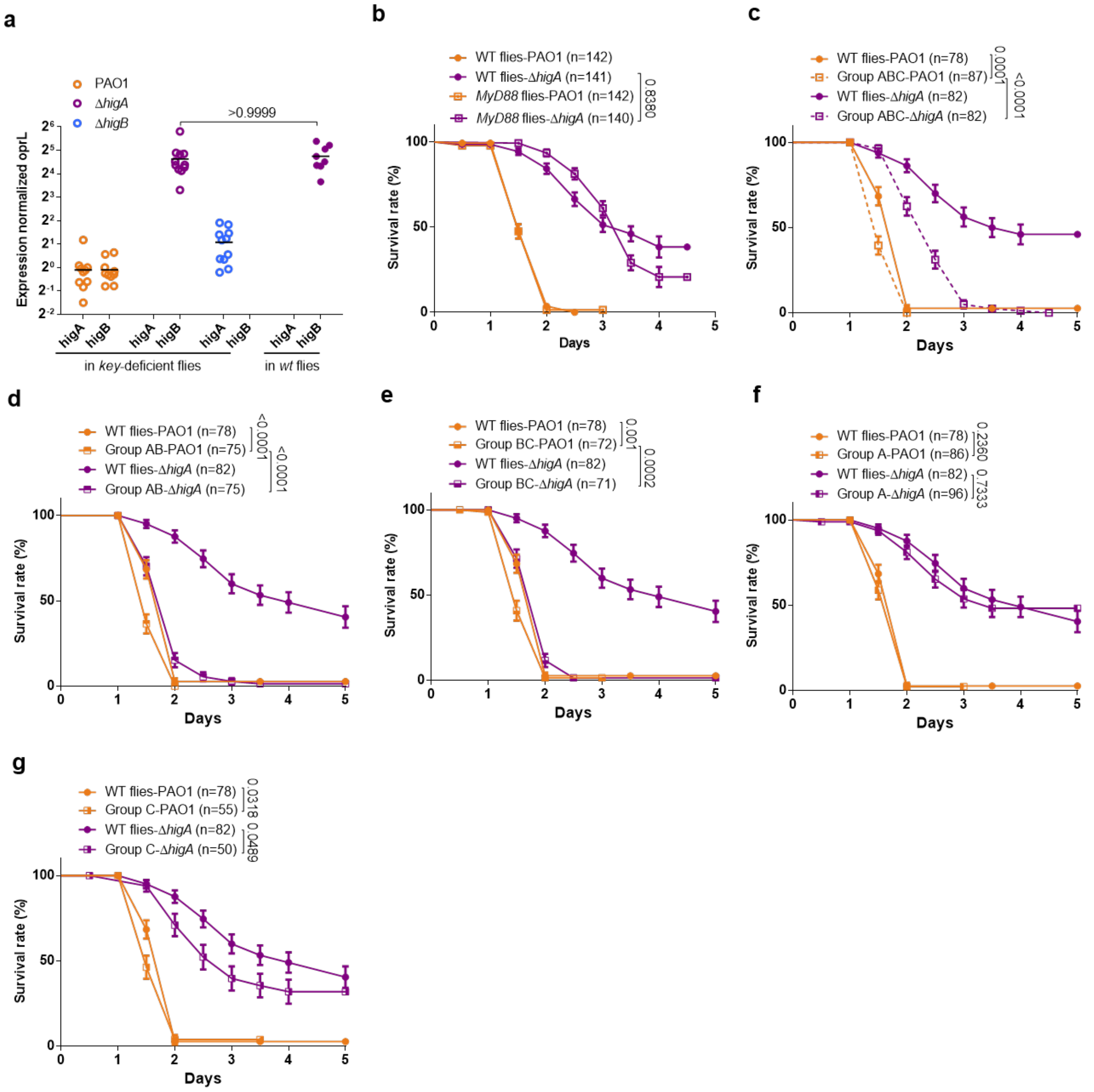

Supplementary Figure 2. Indirect interaction between the *P. aeruginosa* HigB toxin and specific antimicrobial peptide classes in *Drosophila*

a Transcription level of *higA* and *higB* in PAO1 infecting wild-type (*wt*) or *key* flies. **b** Survival of *MyD88*-deficient flies challenged by Δ*higA*. **c** Survival of Group ABC AMP genes-deficient flies challenged by Δ*higA*. **d** Survival of Group AB AMP genes-deficient flies challenged by Δ*higA*. **e** Survival of Group BC AMP genes-deficient flies challenged by Δ*higA*. **f** Survival of Group A AMP gene-deficient flies challenged by Δ*higA*. **g** Survival of Group C AMP genes-deficient flies challenged by Δ*higA*. Group A includes only *defensin*, Group B includes *Diptericins*, *Attacins* and *Drosocin/Buletin*, Group C includes *Drosomycin*

and *Metchnikowin*. Bars represent mean (a), or the standard error of the mean (b-g). Experiments were repeated three times (a-g; pooled data). Statistical analysis was done by two-tailed students's t-test in (a) and Logrank (Mantel-Cox test) in (b-g).

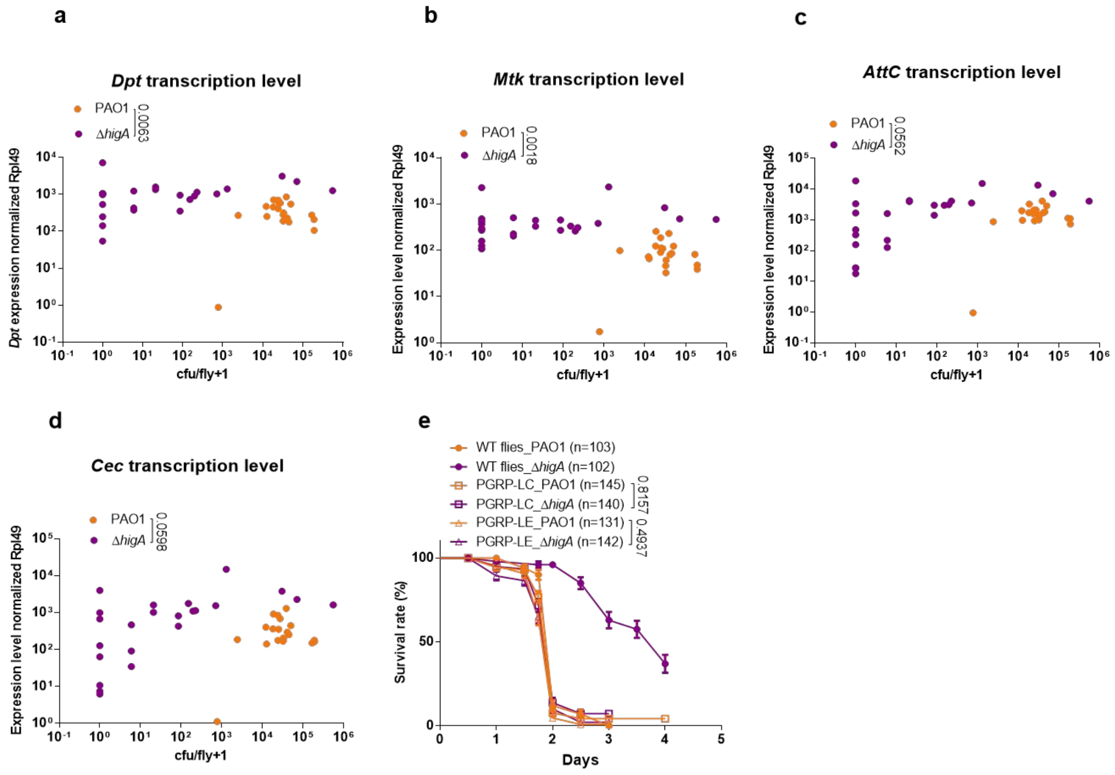

Supplementary Figure 3. The *P. aeruginosa* HigB toxin displays at best mild regulatory effects on the transcription levels of *Drosophila* antimicrobial peptide genes

a-d Transcription level of antimicrobial peptide genes in single fly in correspondence to their bacterial load: *Diptericins*, *Metchnikowin*, *Attacins* and *Cecropin*. **e** Survival of *PGRP-LE*- and *PGRP-LC*-deficient flies challenged by Δ*higA*. Experiments were repeated three times (a-e; pooled data; PAO1 (n=20); Δ*higA*(n=24))). Statistical analysis was done by two-tailed students's t-test in (a-d) and Logrank (Mantel-Cox test) in (e).

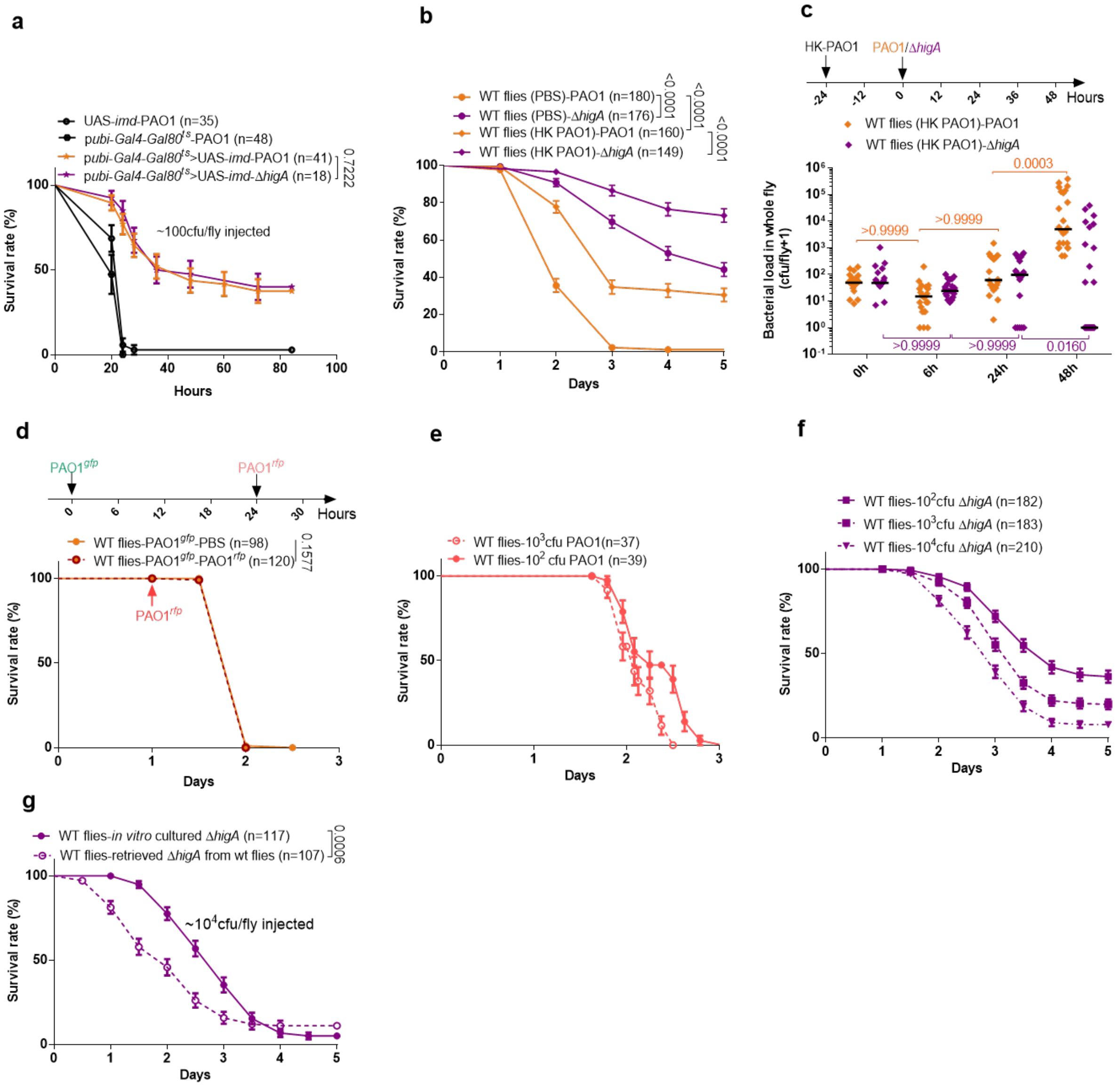

Supplementary Figure 4. Sensitivity and fitness of *P. aeruginosa* exposed to host antimicrobial peptides

a Survival of *imd*-overexpressing flies challenged by PAO1 and Δ*higA*. **b** Survival of pre-immunized flies by heat-killed bacteria challenged with PAO1 and Δ*higA*. **c** Bacterial load corresponding to (b). **d** Survival of flies challenged by secondary *P. aeruginosa* PAO1 injection. **e** Survival of flies challenged by different doses of PAO1. **f** Survival of flies challenged by different doses of Δ*higA*. **g** Virulence of Δ*higA* retrieved from *wt* flies. Bars represent the median (c). Experiments were repeated three times (a-d, g; pooled data; c(n=24)), once in (e-f). Statistical analysis was done by Logrank (Mantel-Cox test) in (a-b, d-g), and by ANOVA and Tukey's multiple comparisons test in (c).

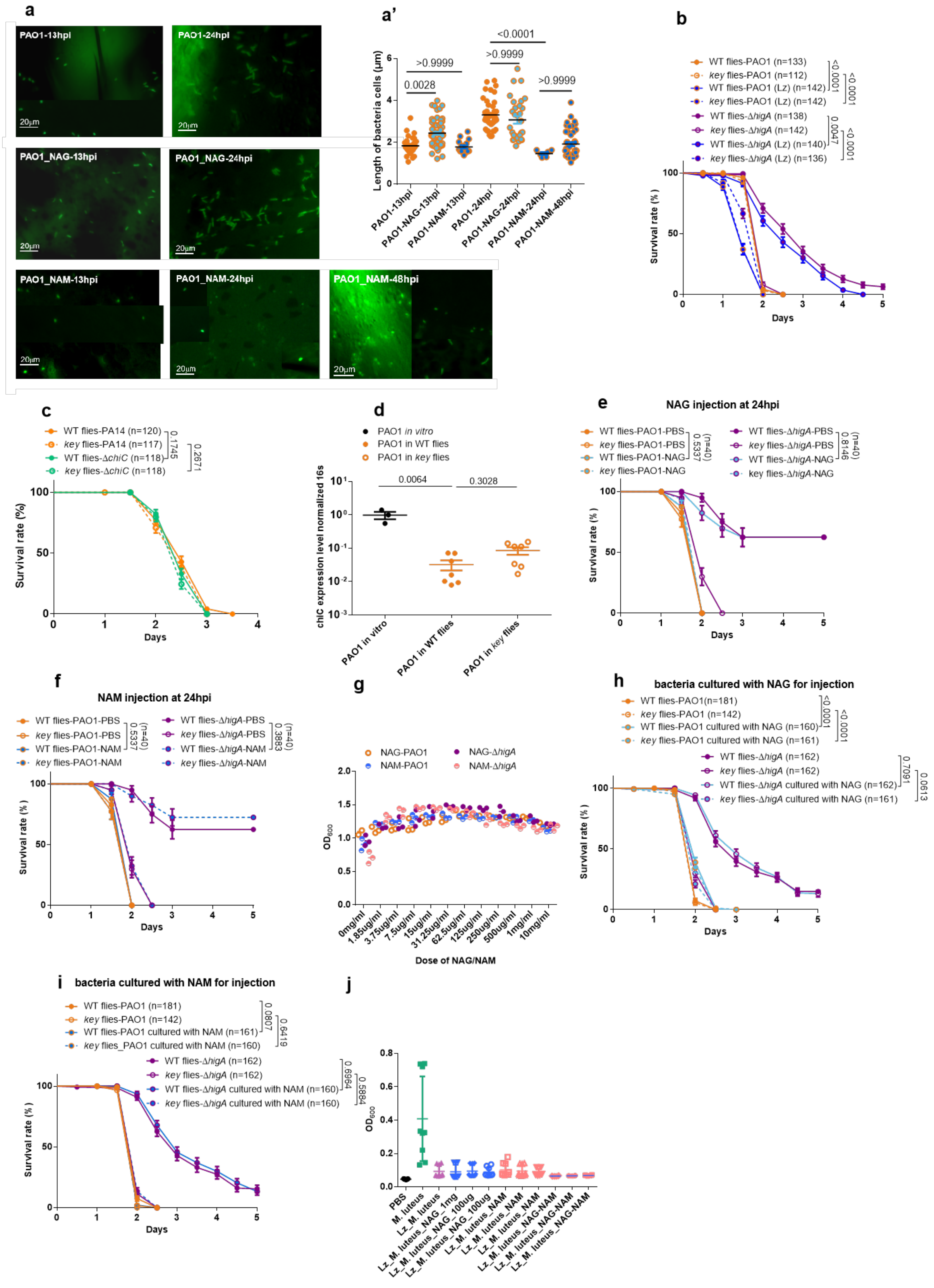

Supplementary Figure 5. Opposite effects of N-acetylglucosamine and N-acetylmuramic acid on *P. aeruginosa* priming

a Morphology of *P. aeruginosa* PAO1 with NAG or NAM treatment in *Drosophila* visualized by GFP-labeled bacteria examined by fluorescence microscopy. Because of the limited number of bacterial cells in most visual field, some of the figures (NAM treatment panels) are composites integrated from different visual fields. **a'** Quantification of bacterial cells length in (a). **b** Survival of flies challenged by PAO1 and Δ*higA* pre-treated with lysozyme. Lz, lysozyme. **c** Survival of flies challenged by PA14 and PA14 Δ*chiC* mutant (PA14 appears to behave similarly to PAO1). **d** Transcription level of *chiC in vitro* and *in vivo*. **e** Survival of flies challenged with PAO1 and Δ*higA* and then treated with NAG at 24hpi. **f** Survival of flies challenged with *P. aeruginosa* PAO1 and Δ*higA* and then injected with NAM at 24hpi. **g** Effect of NAG/NAM on PAO1 and Δ*higA* growth *in vitro* (data in triplicates). **h** Survival of flies challenged with PAO1 and Δ*higA* cultured in the presence of NAG. **i** Survival of flies challenged with PAO1 and Δ*higA* cultured in the presence of NAM. **j** Effect of NAG/NAM on the enzymatic activity of lysozyme. NAG, N-acetylglucosamine; NAM, N-acetylmuramic acid. Bars represent the mean (a', d, j), or the standard error of the mean (b-c, e-f, h-i). Experiments were repeated three times (a-c, e-f, h-i; pooled data； a': PAO1-13hpi (n=30); PAO1-NAG-13hpi (n=67); PAO1-NAM-13hpi (n=16); PAO1-24hpi (n=44); PAO1-NAG-24hpi (n=31); PAO1-NAM-24hpi (n=6); PAO1-NAM-48hpi (n=44).), twice in (d, j; pooled data; d: PAO1 *in vitro* (n=3); PAO1 in WT flies (n=8); PAO1 in *key* flies (n=8). g (n=3). j (n=6-9)), twice in (d, j; pooled data) once in (g). Statistical analysis was done by Logrank (Mantel-Cox test) in (b-c, e-f, h-i), by Kruskal-Wallis with Dunn's post-hoc test in (a', d).

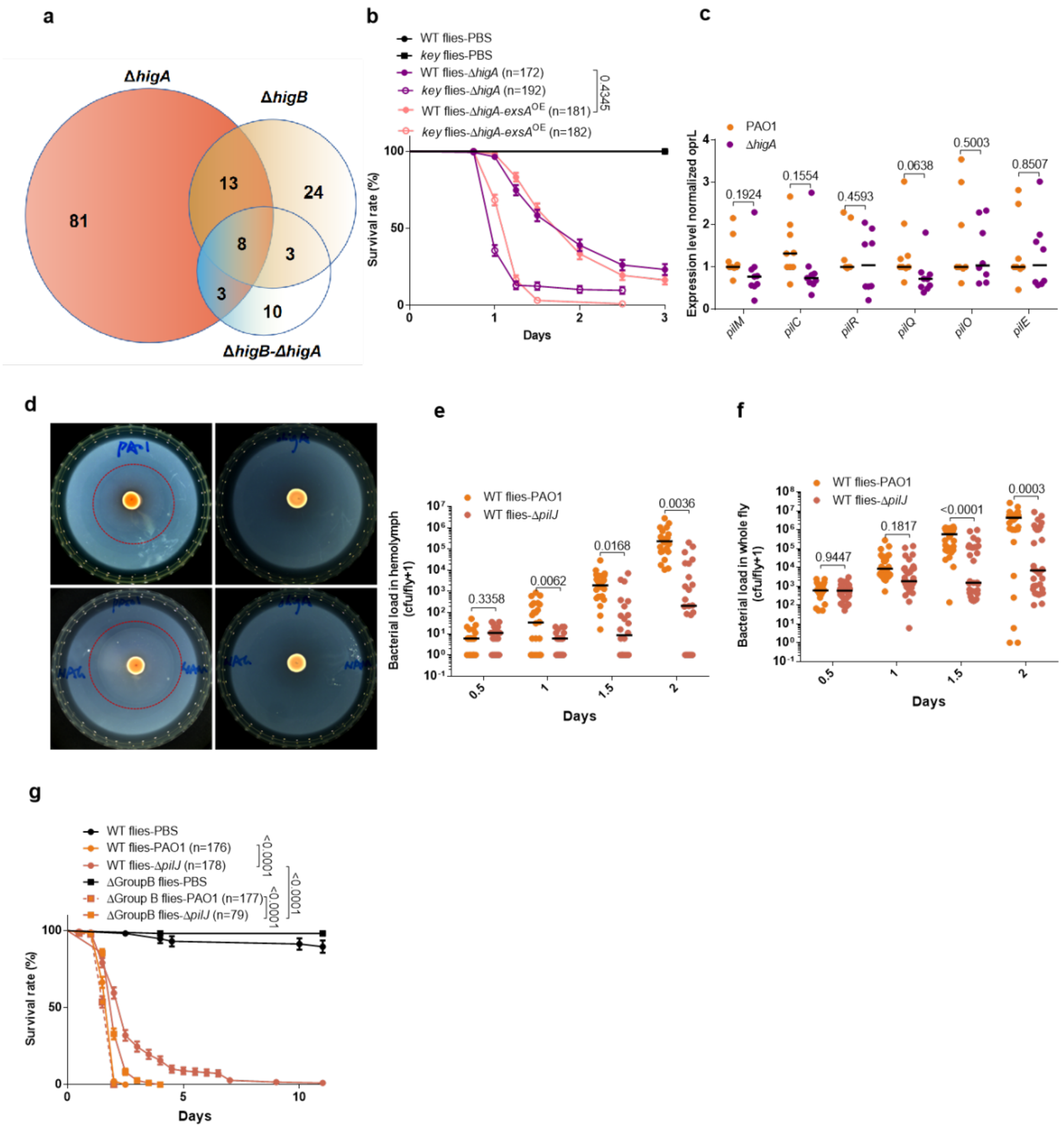

Supplementary Figure 6. Proteomic analysis reveals that type IV pili contribute to *P. aeruginosa* priming in *Drosophila*

a Venn map of differential expression proteins based on proteomics for the set of *higBA* mutants. **b** Survival of flies challenged by Δ*higA* overexpressing *exsA*. **c** Transcription level of type IV pili genes in PAO1 and Δ*higA*. **d** Twitching motility assay of *P. aeruginosa* PAO1 and Δ*higA* without or with NAG and NAM treatment. **e** Bacterial load of *P. aeruginosa* PAO1 and Δ*pilJ* in circulating hemolymph. **f** Bacteria load of *P. aeruginosa* PAO1 and Δ*pilJ* in whole flies. **g** Survival of Group B AMP genes-deficient flies challenged by PAO1 and Δ*pilJ*.

Bars represent the standard error of the mean (b, g), the median (c, e-f). Experiments were repeated three times (b-g; pooled data; c (n=7-9); e-f (n=24)), one of the experiments shown in (d), once in (a). Statistical analysis was done by Logrank (Mantel-Cox test) in (b and g), students's t-test in (c, e-f).

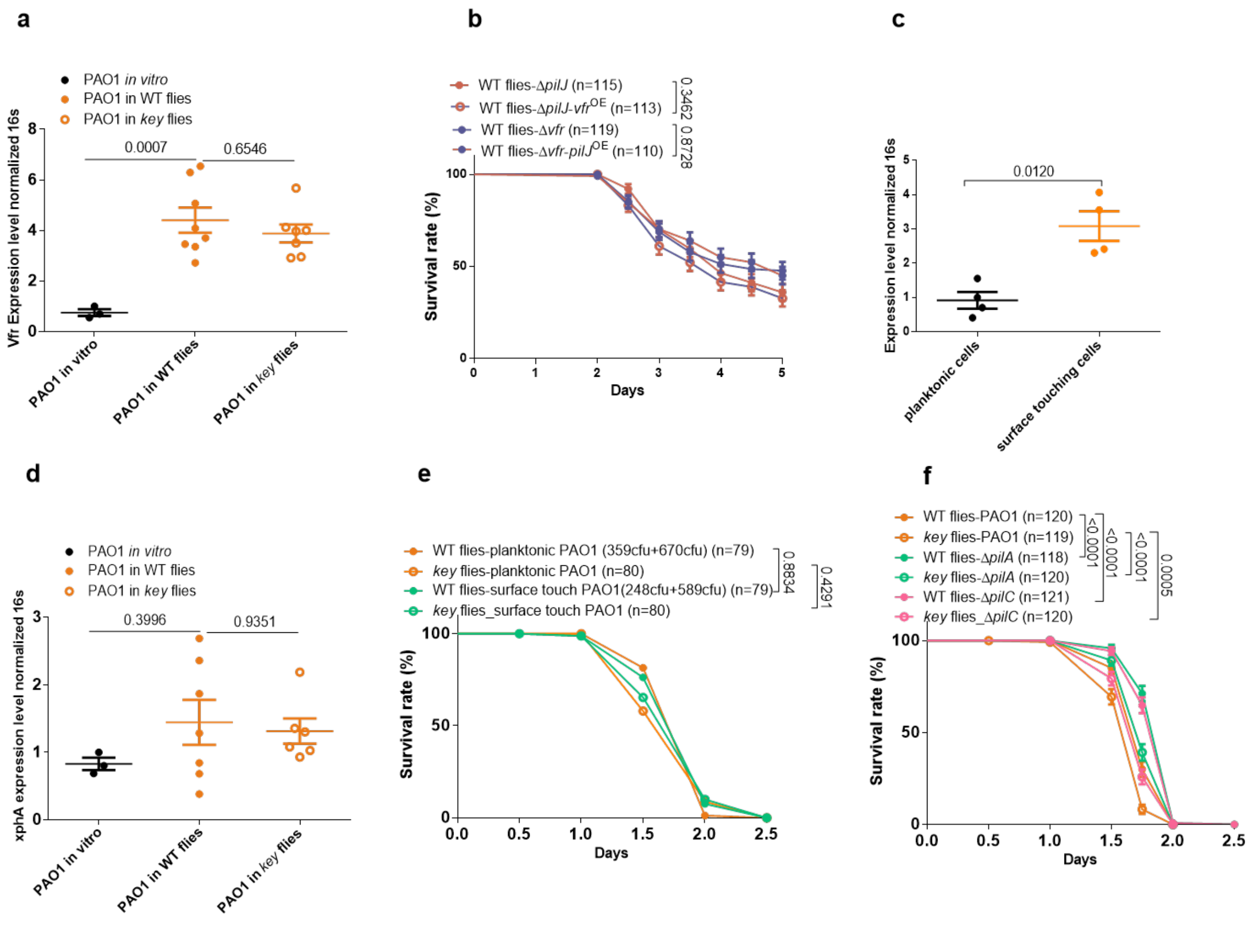

Supplementary Figure 7. Surface sensing signaling is not required for *P. aeruginosa* priming in *Drosophila*

a Transcription level of *vfr in vitro* and *in vivo*. **b** Survival of flies challenged by Δ*pilJ* overexpressing virulence regulator gene *vfr* and *vice versa*. **c** Transcription level of *xphA* in planktonic cells and surface touching cells *in vitro*. **d** Transcription level of *xphA in vitro* and *in vivo*. **e** Survival of flies challenged by planktonic cells and surface touching cells. This experiment was performed twice. **f** Survival of flies challenged by Δ*pilA* and Δ*pilC*. Bars represent the mean (a, c-d) or the standard error of the mean (b, e-f). Experiments were repeated three times (c, f; pooled data), twice in (a, c, d-e; pooled data; a: PAO1 *in vitro* (n=3); PAO1 in WT flies (n=8); PAO1 in *key* flies (n=8). c(n=4). d: PAO1 in vitro (n=3); PAO1 in WT flies (n=7); PAO1 in *key* flies (n=6)). Statistical analysis was done by ANOVA and Tukey's multiple comparisons test in (a,d), by Logrank (Mantel-Cox test) in (b, e-f), and by t-test in (c).

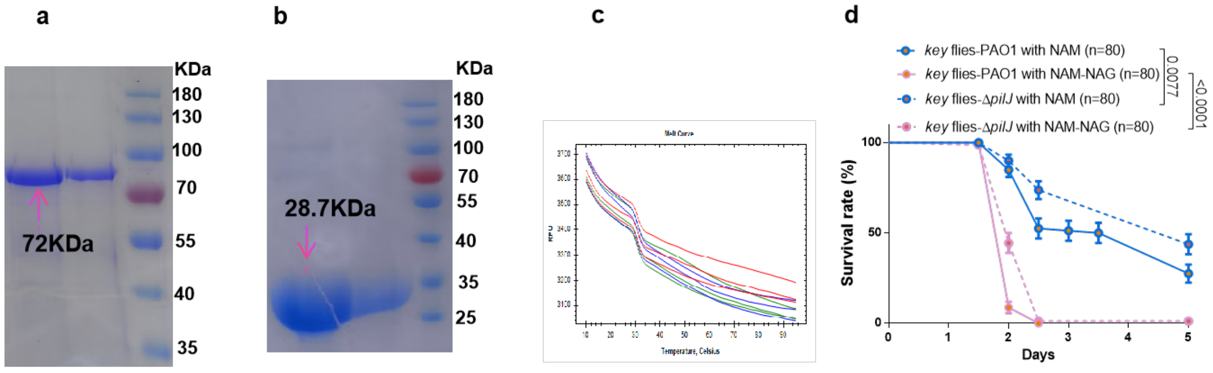

Supplementary Figure 8. Expression and purification of PilJ and its tandem LBD for thermal shift assay

a Purified full length recombinant PilJ protein. **b** Purified truncated recombinant PilJ protein that contains just the two tandem ligands binding domains. **c** Thermal shift assay of recombinant PilJ protein incubated without (red lines) or with NAG (blue lines) or NAM (green lines). **d** Survival of flies challenged by Δ*pilJ* with NAM or NAM-NAG co-injection in *key* flies (complementary to Fig. 5j). Bars represent standard error of the mean (d). Experiments performed three times (c, d), representative data shown in (c), pooled in (d). Statistical analysis was done by Logrank (Mantel-Cox test) in (d).

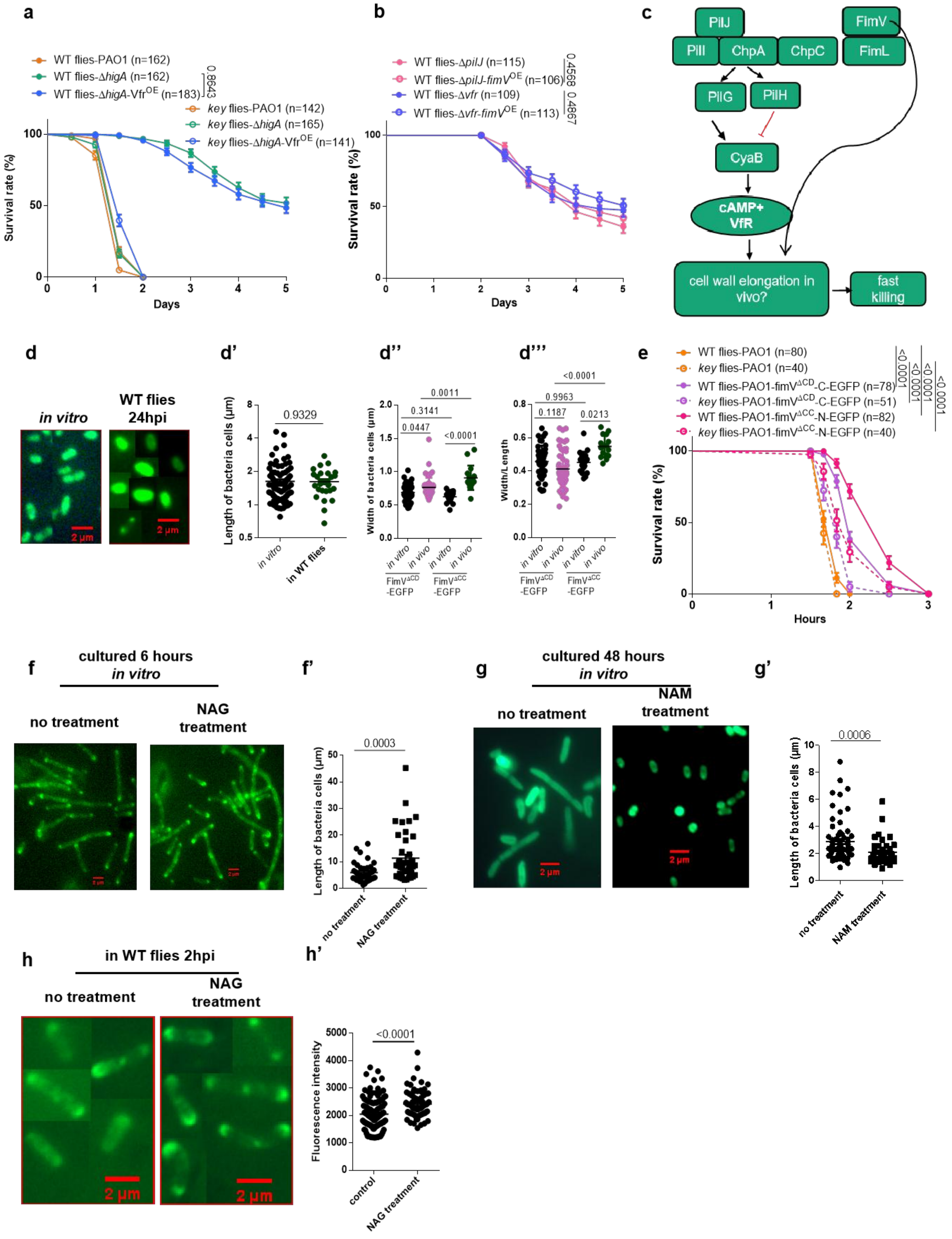

Supplementary Figure 9. Role of the polar protein FimV in *P. aeruginosa* priming in *Drosophila*

a Survival of flies challenged by Δ*higA* overexpressing virulence regulator gene *vfr*. **b** Survival of flies challenged by Δ*pilJ or* Δ*vfr* overexpressing *fimV*. **c** Hypothetical scheme of

Pil-Chp signaling on *P. aeruginosa* priming *in vivo*. **d** Morphology *in vitro* and *in vivo* of PAO1 overexpressing the truncated FimV missing the coiled-coil domain. For the limited number of bacterial cells in some visual fields, all figures *in vivo* are composite assemblies from different visual fields. **d'-d''** Quantification of bacterial shape in for cell length (d'), for width (d''), for the ratio between width and length (d'''). **e** Survival of flies challenged by PAO1 overexpressing the truncated *fimV* missing the coiled-coil domain or the cytoplasmic domain. **f** Morphology of *fimV-EGFP* overexpressing bacteria treated or not with NAG at 6 hours post inoculation. **f'** Quantification of bacteria cell length in (f). **g** Morphology of *fimV-EGFP* overexpressing bacteria treated or not with NAM at 48 hours post inoculation. **g'** Quantification of bacterial cell length in (g). **h** Localization of FimV-GFP in bacteria retrieved from the hemolymph of flies injected or not with NAG two hours earlier. **h'** Quantification of the GFP fluorescence at the bacterial poles using Microbe J. Bars represent standard error of the mean (a-b, e) or the mean (d'-d''', f', g', h'). Experiments were repeated three times (a-b, e-g; pooled data; f': no treatment (n=41), NAG treatment (n=47); g': no treatment (n=75), NAG treatment (n=41)), twice in (d-d'''; pooled data; d': in vitro (n=90); in WT flies (n=27); d''-d''': FimV$^{\Delta CD}$-EGFP-*in vitro* (n=42); FimV$^{\Delta CD}$-EGFP-*in vivo* (n=53); FimV$^{\Delta CC}$-EGFP-*in vitro* (n=34); FimV$^{\Delta CC}$-EGFP-*in vivo* (n=31)), once in (h). Statistical analysis was done by Logrank (Mantel-Cox test) in (a-b, e), student's t-test in (d', h'), ANOVA and Tukey's multiple comparisons test in (d''-d''').

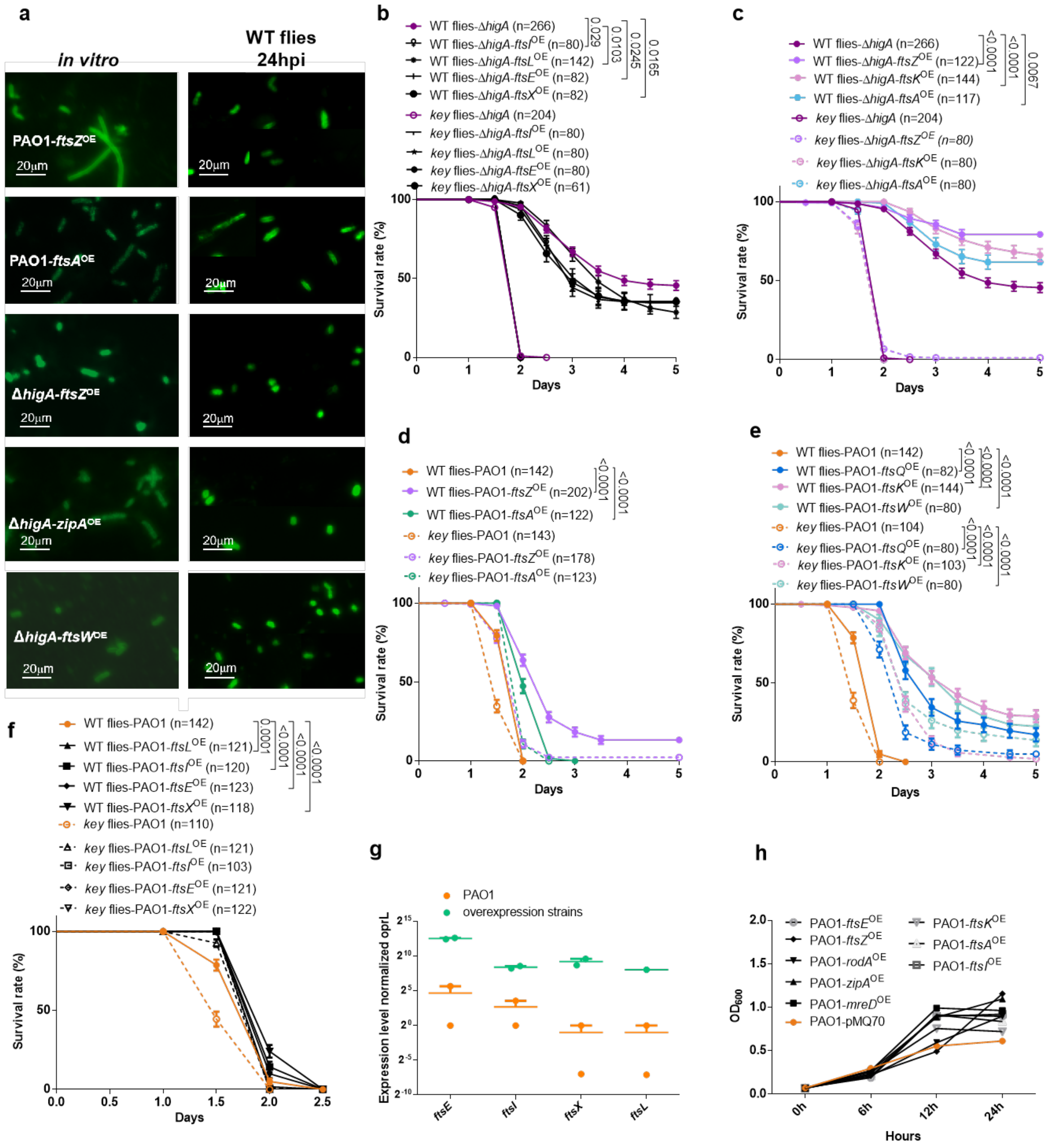

Supplementary Figure 10. Effect of the overexpression of cell division genes on *P. aeruginosa* priming in *Drosophila*

a Morphology of *P. aeruginosa* PAO1 and Δ*higA* overexpressing cell division genes *in vitro* and *in vivo* visualized by O5 antibody staining under fluorescence microscope. Because of the limited number of bacterial cells in most visual fields, all figures *in vivo* are composite assemblies integrated from different visual fields. **b-c** Survival of flies challenged by Δ*higA* overexpressing cell division genes. **d-f** Survival of flies challenged by wild-type PAO1

overexpressing cell division genes. **g** Transcription level of cell division genes in overexpressing strains. Please, note that we cannot formally exclude that the overexpressed proteins are not stable. **h** Growth characteristic of PAO1 overexpressing cell division genes *in vitro*. Bars represent standard error of the mean (b-f, h), median (g, n=2). Experiments were repeated three times (a-f; pooled data), once in (g-h). Statistical analysis was done by Logrank (Mantel-Cox test) in (b-f).

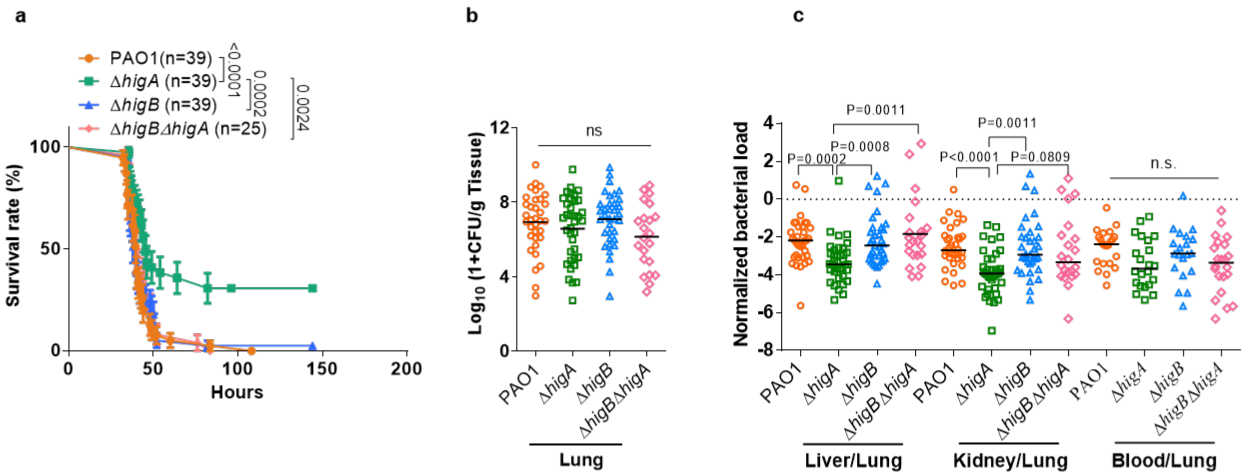

Supplementary Figure 11. Δ*higA* mutants are less virulent in a murine acute lung infection model

a Survival of mice challenged by *higBA* mutants. **b** Bacterial titer of *higBA* mutants in lung tissue. **c** Bacterial titer of *higBA* mutants in liver, kidney and blood tissues after respiratory infection (inhalation of a concentrated bacterial solution). Bars represent the standard error of the mean (a) or the mean (b-c). Experiments were repeated three times (a-c; pooled data; b-c: PAO1 (n=34); Δ*higA* (n=37); Δ*higB* (n=35); Δ*higBA* (n=22)). Statistical analysis was done by Logrank in (a), by t test in (b-c).
