# Supplementary material for: N-acetyl-glucosamine primes Pseudomonas aeruginosa for virulence through a type IV pili/cAMP-mediated morphology transition
Source: Nat Commun. 2025 Oct 24;16:9405. doi: 10.1038/s41467-025-64071-0 (PMC12552656; doi:10.1038/s41467-025-64071-0)
Supplement: Supplementary file 2 — Description of Additional Supplementary File [file 41467_2025_64071_MOESM2_ESM.pdf]

Description of Additional Supplementary Files

Supplementary Data 1: List of differentially-expressed proteins in P. aeruginosa mutants of the higBA operon

Supplementary Data 2: Reagents and resource

Supplementary Data 3: List of primers used

Supplementary Data 4: List of reagents and software used
